# Validation of a deep learning algorithm for bone age estimation among patients in the city of São Paulo, Brazil

**DOI:** 10.1590/0100-3984.2023.0056-en

**Published:** 2023

**Authors:** Augusto Sarquis Serpa, Abrahão Elias Neto, Felipe Campos Kitamura, Soraya Silveira Monteiro, Rodrigo Ragazzini, Gustavo Antunes Rodrigues Duarte, Lucas André Caricati, Nitamar Abdala

**Affiliations:** 1 Escola Paulista de Medicina da Universidade Federal de São Paulo (EPM-Unifesp), São Paulo, SP, Brazil; 2 Dasa, São Paulo, SP, Brazil; 3 Ionic Health, São José dos Campos, SP, Brasil

**Keywords:** Artificial intelligence, Machine learning, Deep learning, Bone development, Growth., Inteligência artificial, Aprendizado de máquina, Aprendizado profundo, Desenvolvimento ósseo, Crescimento.

## Abstract

**Objective:**

To validate a deep learning (DL) model for bone age estimation in individuals in the city of São Paulo, comparing it with the Greulich and Pyle method.

**Materials and Methods:**

This was a cross-sectional study of hand and wrist radiographs obtained for the determination of bone age. The manual analysis was performed by an experienced radiologist. The model used was based on a convolutional neural network that placed third in the 2017 Radiological Society of North America challenge. The mean absolute error (MAE) and the root-mean-square error (RMSE) were calculated for the model versus the radiologist, with comparisons by sex, race, and age.

**Results:**

The sample comprised 714 examinations. There was a correlation between the two methods, with a coefficient of determination of 0.94. The MAE of the predictions was 7.68 months, and the RMSE was 10.27 months. There were no statistically significant differences between sexes or among races (*p* > 0.05). The algorithm overestimated bone age in younger individuals (*p* = 0.001).

**Conclusion:**

Our DL algorithm demonstrated potential for estimating bone age in individuals in the city of São Paulo, regardless of sex and race. However, improvements are needed, particularly in relation to its use in younger patients.

## Abstract (pt)

**Objetivo:**

Validar em indivíduos paulistas um modelo de aprendizado profundo (*deep learning* - DL) para estimativa da idade óssea, comparando-o com o método de Greulich e Pyle.

**Materiais e Métodos:**

Estudo transversal com radiografias de mão e punho para idade óssea. A análise manual foi feita por um radiologista experiente. Foi usado um modelo baseado em uma rede neural convolucional que ficou em terceiro lugar no desafio de 2017 da Radiological Society of North America. Calcularam-se o erro médio absoluto (*mean absolute error* - MAE) e a raiz do erro médio quadrado (*root mean-square error* - RMSE) do modelo contra o radiologista, com comparações entre sexo, etnia e idade.

**Resultados:**

A amostra compreendia 714 exames. Houve correlação entre ambos os métodos com coeficiente de determinação de 0,94. O MAE das predições foi 7,68 meses e a RMSE foi 10,27 meses. Não houve diferenças estatisticamente significantes entre sexos ou raças (*p* > 0,05). O algoritmo superestimou a idade óssea nos mais jovens (*p* = 0,001).

**Conclusão:**

O nosso algoritmo de DL demonstrou potencial para estimar a idade óssea em indivíduos paulistas, independentemente do sexo e da raça. Entretanto, há necessidade de aprimoramentos, particularmente em pacientes mais jovens.

## INTRODUCTION

Accurate determination of bone age plays a vital role in monitoring bone development, acting as a reliable indicator of biological age and growth prognosis^**(^1^)**^. There are several manual methods of bone age estimation that use radiographs of various parts of the body^**(^2^)**^. However, the hand and wrist are most often chosen, because of the presence of multiple ossification centers, simplicity of the technique, adequate radiation safety, and low cost of the procedure^**(^1^)**^. The use of the left limb is recommended for a number of reasons, including the fact that most people are right-handed and there is therefore a greater chance of bone injuries on the right side^**(^1^)**^.

Among the radiographic methods used in the assessment of bone age, that devised by Greulich and Pyle^**(^3^)**^ is the most widely used^**(^2^)**^. Their method involves the analysis of the ossification centers of the left hand and wrist in comparison with a standard image atlas. However, it was originally developed in the 1950s and was based on a population of White individuals in the United States^**(^3^)**^. Therefore, its applicability and precision, when used in other populations, have been questioned^**(^4^)**^. In addition, there is controversy in the literature regarding its reproducibility, with significant discrepancies among the results of studies that aimed to evaluate the intraobserver and interobserver variability for readings^**(^5^)**^. Given this scenario, various automated models that use artificial intelligence (AI) to estimate bone age have been proposed, most of them based on traditional machine learning (ML), BoneXpert being the most widely used^**(^6^,^7^)**^. However, most of the algorithms were based on populations in the United States or western Europe, and few studies have taken the ethnic and socioeconomic particularities of the individuals into consideration in the analysis of the results^**(^6^)**^.

To date, there have been no studies evaluating the performance of bone age estimation algorithms in the population of Brazil. Therefore, the aim of this study was to validate, in children and adolescents in the city of São Paulo, Brazil, the predictions of a model based on deep learning (DL), a subtype of ML, for estimating bone age, comparing the results obtained with an analysis carried out by a trained radiologist using the Greulich-Pyle method. Such local validation is essential to ensure the accuracy and clinical relevance of these AI models before their large-scale implementation in clinical settings in Brazil.

## MATERIALS AND METHODS

This was a cross-sectional study of radiographs of the left hand and wrist obtained at our facility, recorded as examinations for the determination of bone age. The study was approved by the research ethics committee, on the basis of the research project “Development of medical imaging databases to promote research and challenges in machine learning in the field of radiology”.

A database of radiographs obtained between 2018 and 2022 was created. The inclusion criterion was having an available radiology report describing bone age. All examinations were reported by a radiologist with three years of experience in bone age determination by the Greulich-Pyle method. Bilateral examinations were excluded, as were examinations of other parts of the body that were incorrectly recorded, those performed with inappropriate technique or positioning, those in which there were peripheral catheters, and those in which there were bone deformities that hindered the analysis. A convenience sample was used because there is no universally accepted sample calculation method for DL models. The examinations were anonymized with specific Radiological Society of North America (RSNA) software (RSNA Anonymizer), the download and source code of which are available at http://mirc.rsna.org/download/Anonymizer-installer.jar.

The images were uploaded to cloud-based medical image annotation software (MD.ai; MD.ai, Inc., New York, NY, USA). Through this, it was noted which radiographs met one or more of the exclusion criteria, for later elimination from the study. Data regarding the age, sex, race, chronological age, and reported bone age were also recorded for each patient by consulting the medical records. The five races proposed by the classification of the Brazilian Institute of Geography and Statistics^**(^8^)**^, according to the last published census, were as follows: Asian, White, Indigenous, Mixed, and Black.

**Figure 1:**
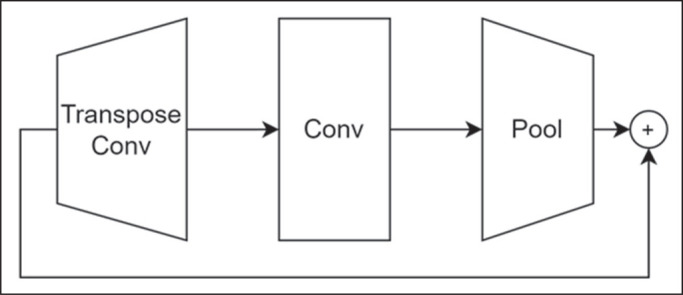
Architecture of the ice module, the basic block of the model used, which consists of a transposed convolution (Transpose Conv) layer followed by a convolution (Conv) layer and a pooling (Pool) layer, as well as a shortcut through a residual connection.

After the data had been collected and annotated, inferential analysis of the examinations was performed by using a DL model based on a convolutional neural network (Figure 1) developed by the Federal University of São Paulo in partnership with the Federal University of Goiás. In the training phase for the algorithm, the database was divided into five subsets and cross-validated. The final prediction involves the arithmetic mean of the four models that had the best individual result. The hyperparameters were as follows: initial learning rate, 10^-4^; batch size, 16; and epochs, 100. The Adam optimizer was used. As preprocessing of the images, all pixels are divided by 255, so that they are in the interval [0, 1], after which they are normalized by the mean and standard deviation of each examination. The image is then resized to 550 × 550 pixels, preserving the original proportions, and, if necessary, padding with zeros is performed on the edges of the image. Those preprocessing steps were also used for all radiographs included in the present study. In the training phase, data amplification was also carried out in a proportion of the examinations, with modifications such as a rotation of ±30°, inversion on the horizontal axis, and a zoom of ±10%. This model was previously trained and tested with the AI competition database of the RSNA in 2017, having ranked third among 260 participating teams from all over the world, with a mean absolute error (MAE) of 4.38 months^**(^9^)**^.

The next step was a comparative analysis versus the radiologist report. The absolute errors of the algorithm result, in relation to the conventional reading, were calculated for each patient, and the result was expressed in months. The MAE was calculated by determining the sum of absolute errors and dividing it by the total number of examinations. This metric is widely used to evaluate the performance of AI algorithms that involve predictions of numerical variables. Its advantage over other similar metrics, such as the root-mean-square error (RMSE), is that it is not subject to variations in the distribution of error magnitude and sample size^**(^10^)**^. However, because other studies of this topic use the RMSE, we also calculated that metric for the sample as a whole, in order to allow comparative analyses with such studies.

Because the variables of interest did not have a normal distribution, descriptive analyses were also carried out using median and interquartile range (interquartile range). To detect points outside the curve, the equation *median* + 1.5 × *interquartile range* was employed. Comparative statistical analyses of results by sex and age group were performed with the Mann-Whitney test. For comparisons among races, the Kruskal-Wallis test was applied for all groups. In addition, linear regression was performed to detect differences between the reported bone age and the algorithm’s prediction, with calculation of the Pearson correlation coefficient and coefficient of determination. A Bland-Altman plot was constructed to study the non-absolute error, which preserves the information if the model overestimated or underestimated the bone age in comparison with the estimation made by the radiologist.

The study was carried out with the Python programming language, version 3.0^**(^11^)**^, using the Pandas^**(^12^)**^ and SciPy^**(^13^)**^ libraries for statistical analyses. To create the graphs, the MatPlotLib^**(^14^)**^ and Seaborn^**(^15^)**^ libraries were used. For algorithm inference, the PyTorch^**(^16^)**^ and NumPy^**(^17^)**^ packages were used. In all conclusions obtained by inferential analyses, a significance level of 5% (*p* ≤ 0.05) was adopted.

## RESULTS

**Figure 2:**
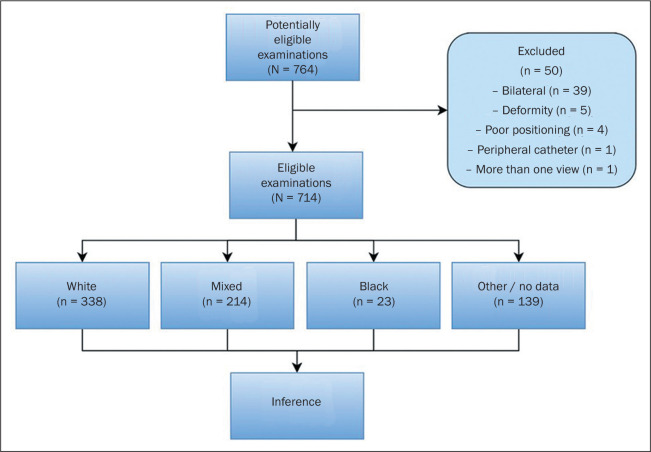
Study flow chart.

A total of 764 examinations met the inclusion criteria and were eligible. Of those, 50 were eliminated because they met one of the exclusion criteria, leaving 714 examinations (Figure 2). The demographic data of the patients who underwent those 714 examinations are presented in Table 1. Ages ranged from 1 year and 3 months to 19 years and 10 months, and only six patients were under 3 years of age. For 137 patients, there was no information about race, and the corresponding radiographs were excluded from the analyses of that independent variable. There was only one Asian patient and one Indigenous patient, both of whom were also excluded from those analyses because of an insufficient number of cases.

**Table 1:** General characteristics of the study sample.

Variable	(N = 714)
Sex, n (%)	
Female	369 (51.68)
Male	345 (48.32)
Chronological age (years), median (IQR)	10.79 (8.27-13.33)
Bone age (years), median (IQR)	11 (8.83-13.5)
Race, n (%)	
White	338 (47.34)
Mixed	214 (29.97)
Black	23 (3.22)
Asian	1 (0.14)
Indigenous	1 (0.14)
No data	137 (19.19)

**Figure 3:**
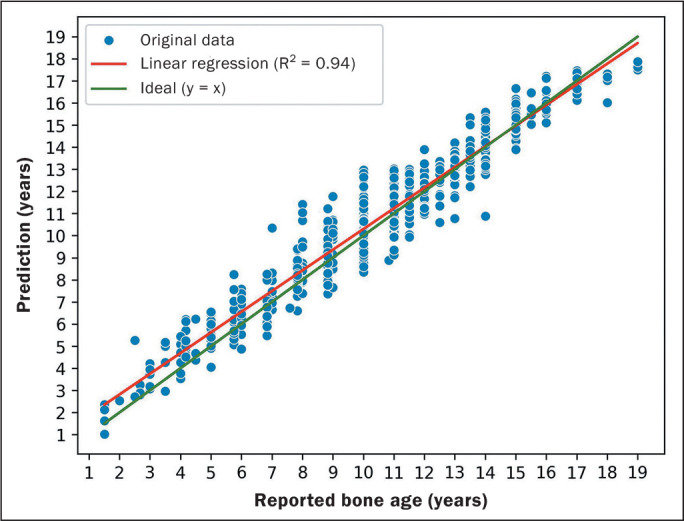
Graph of predictions in relation to reported bone age.

**Figure 4:**
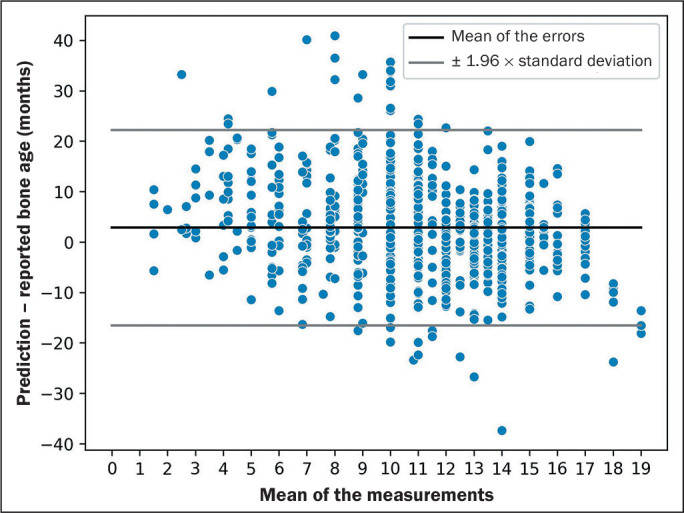
Bland-Altman plot of the error in relation to the mean of the manual measurements and the algorithm.

In the study of the correlation between the bone age reported by the radiologist and the algorithm prediction, linear regression was performed with a line that was close to the ideal line, which would be the case in which all of the predictions of the model were correct (y = x). The Pearson correlation coefficient was 0.97 and the determination coefficient was 0.94 (Figure 3). The analysis of the linear correlation and of the Bland-Altman plot (Figure 4), which illustrates the non-absolute error (prediction - reported bone age), suggested a tendency for the algorithm to overestimate bone age in younger people.

**Figure 5:**
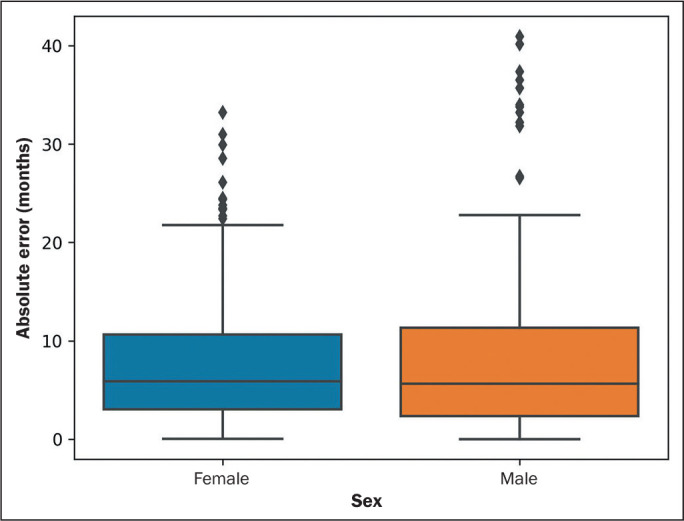
Box and whisker plot of absolute errors, by sex, in the study sample. Diamonds indicate outliers.

**Figure 6:**
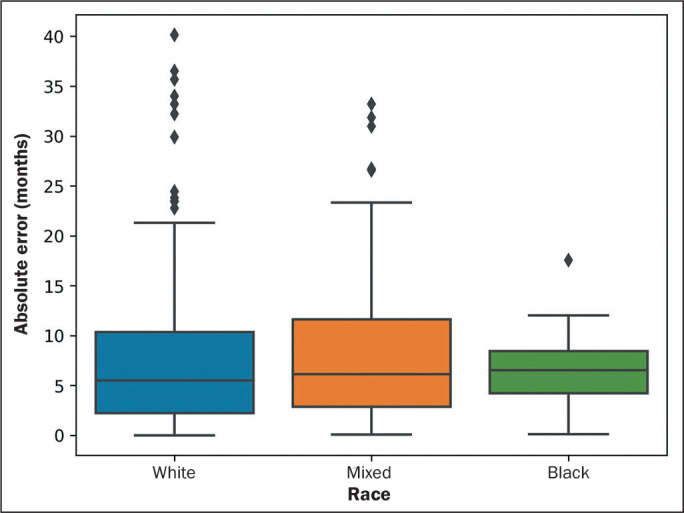
Box and whisker plot of absolute errors in relation to each race in the study sample. Diamonds indicate outliers.

The MAE of the predictions in relation to the reported bone age was 7.68 months for the sample as a whole. The RMSE was 10.27 months (0.86 years). Table 2 describes the MAEs, expressed as medians and interquartile ranges, for all examinations and broken down by sex, race, and age group, together with the respective *p*-values for the inferential analyses between and among the groups. The data were divided at the 50th percentile for chronological age, to test the hypothesis that the model overestimated bone age in younger individuals, which was confirmed. The comparisons between sexes and among races revealed no statistically significant differences (Figures 5 and 6). In the interquartile range analysis, there were 19 points outside the curve (Figures 5 and 6).

**Table 2:** Analysis of the overall MAE, by sex, race, and age, in months.

Variable	MAE	Median	IQR	*P*
Sex (N = 714)				0.*5*75
Female	7.55	5.88	3.05-10.65	
Male	7.82	5.64	2.36-11.34	
Race (n = 575)				0.368^†^
White	7.25	5.50	2.21-10.36	
Mixed	7.85	6.13	2.86-11.61	
Black	6.32	6.52	4.22-8.46	
Age^*^ (N = 714)				0.001^‡^
≤ 10,79 years	8.43	6.42	3.23-11.87	
> 10,79 yeras	6.41	5.16	2.19-9.62	

* 50th percentile for chronological age.

† Kruskal-Wallis multiple comparison test for the three groups.

‡ Statistically significant.

## DISCUSSION

In this study, we sought to validate a DL algorithm for calculating bone age based on radiographs of the hands and wrists of patients followed at our facility. The MAE of the model prediction in relation to the radiologist report was 7.68 months, a value lower than the 9.96 months reported in a recent meta-analysis among studies that used different ML techniques to predict bone age^**(^6^)**^. This indicates that, in a real clinical context, the performance of the algorithm is comparable to or better than those of other proposed models. However, performance in our data was considerably worse relative to the RSNA challenge data, with a MAE of 4.38 months^**(^9^)**^. One possible explanation for that is the fact that the algorithm was trained on populations in the United States, with phenotypes different from those of the population of Brazil. However, it is noteworthy that the test group, in the challenge database, was annotated on the basis of the opinion of six radiologists, which reduces the chance of human error and could, in part, explain this discrepancy between the MAEs.

In our study, the RMSE was 10.27 months (0.86 years) for the sample as a whole. The most widely used and validated algorithm, BoneXpert, based on traditional ML, obtained an RMSE of 0.72 years in its version 2^**(^18^-^20^)**^ and 0.62 years in its version 3^**(^7^)**^, both measured in a test group, independent of the training group, of patients in the city of Tübingen, Germany, and based on the readings of just one radiologist. Despite the poorer performance of our model, it should be borne in mind that the other model was trained on data related to patients of European or North American origin, whose ethnic, socioeconomic, and nutritional characteristics are closer to those of the test group used than to those of our study sample. When we compared our model with one that used DL^**(^21^)**^, also trained on the RSNA database and validated on an external database, we found that the latter performed better than did our algorithm, with an MAE of 5.96 months. However, that model was validated only at centers in the United States, the same country of origin of the examinations on which it was trained, and the annotation was performed by four radiologists, substantially reducing the chance of human error.

The absence of a statistically significant difference between sexes and among races in relation to the absolute error suggests that the model has a uniform performance for boys and girls of different ethnicities, which is a desirable characteristic for clinical application. There have been few studies comparing the performance of bone age algorithms among races^**(^6^)**^. Nevertheless, it is important to note that the low number of Black individuals in the sample might have been insufficient for the statistical test to capture any difference and did not reflect the ethnic distribution of the population of Brazil^**(^22^)**^.

Our automated method overestimated bone age in younger patients. One possible explanation for that finding is the greater variability in bone development among such individuals, which can be difficult for the model to capture^**(^23^)**^. Therefore, improvements in model training could be necessary in order to improve the performance of the algorithm in this age group.

Despite the encouraging results, our study has some limitations that should be considered when interpreting the findings. As previously mentioned, bone age was determined on the basis of the assessment of only one radiologist, which introduces an expected error, given that the interpretation of traditional methods of bone age determination is subjective and can vary between observers^**(^5^,^23^)**^. In addition, our sample had a low number of participants who self-identified as Black, only one who self-identified as Asian, only one who self-identified as Indigenous, and only six who were under the age of three. Those aspects could have reduced the generalizability of our results. Furthermore, there was a significant lack of information about the race of some of the patients. Finally, the lack of detailed information about patient comorbidities is another limitation, because certain medical conditions can influence bone development^**(^23^)**^.

In conclusion, the DL algorithm validated in this study shows promise for estimating bone age in children and adolescents of both sexes and of different races in Brazil. However, it is important to consider its limitations and the need for refinement to improve its clinical applicability, especially in younger patients. In addition, the algorithm should not be seen as a substitute for radiologist assessment, but rather as a complementary tool in the process of determining bone age.
